# Generalized Joint Hypermobility and Anxiety Are Serious Risk Factors for Dysfunctioning in Dance Students: A One-Year Follow-Up Study

**DOI:** 10.3390/ijerph19052662

**Published:** 2022-02-25

**Authors:** Janneke van Die-de Vries, Jeanine Verbunt, Stephan Ramaekers, Patrick Calders, Raoul Engelbert

**Affiliations:** 1 School of Physiotherapie, Amsterdam University of Applied Sciences, 1105 BD Amsterdam, The Netherlands; s.p.j.ramaekers@hva.nl; 2 Department of Rehabilitation, Amsterdam UMC, University of Amsterdam, Meibergdreef 9, Amsterdam Movement Sciences, 1105 AZ Amsterdam, The Netherlands; r.h.h.engelbert@hva.nl; 3 Centre of Expertise Urban Vitality, Faculty of Health, Amsterdam University of Applied Sciences, 1105 BD Amsterdam, The Netherlands; 4 Department of Rehabilitation Medicine, CAPHRI, Functioning and Rehabilitation, Maastricht University Medical Center, 6229 HX Maastricht, The Netherlands; jeanine.verbunt@maastrichtuniversity.nl; 5 Adelante Center of Expertise in Rehabilitation and Audiology, 6432 CC Hoensbroek, The Netherlands; 6 Department of Rehabilitation Sciences, Faculty of Medicine and Health Sciences, Ghent University, 9000 Ghent, Belgium; patrick.calders@ugent.be; 7 Department of Pediatrics, Amsterdam UMC, University of Amsterdam, Emma Children’s Hospital, Meibergdreef 9, 1105 AZ Amsterdam, The Netherlands

**Keywords:** physical performance, joint hypermobility, anxiety, high performing adolescents

## Abstract

Young professional dancers find themselves in a demanding environment. GJH within dancers is often seen as aesthetically beneficial and a sign of talent but was found to be potentially disabling. Moreover, high-performing adolescents and young adults (HPAA), in this specific lifespan, might be even more vulnerable to anxiety-related disability. Therefore, we examined the development of the association between the presence of Generalized Joint Hypermobility (GJH) and anxiety within HPAA with a one-year follow-up. In 52.3% of the HPAA, anxiety did not change significantly over time, whereas GJH was present in 28.7%. Fatigue increased significantly in all HPAA at one year follow-up (respectively, females MD (SD) 18(19), *p* < 0.001 and males MD (SD) 9(19), *p* < 0.05). A significantly lower odds ratio (ß (95% CI) 0.4 (0.2–0.9); *p*-value 0.039) for participating in the second assessment was present in HPAA with GJH and anxiety with a 55% dropout rate after one year. This confirms the segregation between GJH combined with anxiety and GJH alone. The fatigue levels of all HPAA increased significantly over time to a serious risk for sick leave and work disability. This study confirms the association between GJH and anxiety but especially emphasizes the disabling role of anxiety. Screening for anxiety is relevant in HPAA with GJH and might influence tailored interventions.

## 1. Introduction

Young professional dancers find themselves in a highly demanding environment. As their future sporting career and life are determined by their current performance, these high-performing adolescents and young adults (HPAA) often complete extensive training hours [1,2,3]. With expectations and demands placed on the individuals by choreographers, teachers, parents, or themselves [4,5], the pressure to perform in elite sports can be overwhelming, with a possible increase in the risk of injury and psychological overload [6,7]. Research within dance is primarily focused on causes of high injury risk. Self-reported seasonal prevalence of injury ranges from 40% (time-loss) to 92% (all health problems depending on the definition of injury) [8,9,10]. However, two reviews that discuss the possible causes of injury point out that the methodological quality of the included studies is often lacking [11,12]. Factors identified with risk of injury include both physical and psychosocial factors. One of these physical factors is Generalized Joint Hypermobility (GJH), which is highly prevalent with 57% of young high-performing dancers [13,14,15]. Although this factor was found to be potentially disabling, GJH is within the dance community often seen as aesthetically beneficial and a sign of talent. Dance requires complex movements that often extend the normal range of joint movement. GJH can be helpful to excel in sports such as rhythmic gymnastics or dance but can also be accompanied by complaints. Patients with Joint Hypermobility Syndrome (JHS) or Ehlers Danlos syndrome hypermobility type (EDS-HT) show similar complaints such as chronic musculoskeletal pain end soft tissue injury but differ in others, such as skin involvement, positive family history, and recurrent dislocations [16,17]. The diversity within joint hypermobility is presented in the revision of the clinical criteria of EDS in 2017. Soft tissue frailty was added and is now referred to as hypermobile EDS (hEDS). For former EDS-HT patients who do not fulfill the new criteria and JHS patients, the term Hypermobility Spectrum Disorder (G-HSD) is currently used. In GJH, h-EDS, and HSD, disease progression has been barely studied over time and primarily addresses physical functioning [18,19].

The GJH literature does not segregate individuals with GJH from individuals with both GJH and anxiety, although they underline the association between the two, anxiety and GJH [19,20]. Psychosocial complaints within the disease progression are proposed from adulthood based on cross-sectional observations by Castori et al. [21].

Recently, we found in a cross-sectional study that HPAA, a group of young professional dancers with the combination of GJH and anxiety, were showing significantly decreased physical and psychosocial functioning, illustrated by a decreased workload, increased fatigue, and pain catastrophizing [22]. In a cross-sectional study, we confirmed the association between GJH and anxiety but especially emphasized the disabling role of anxiety. The presence of GJH alone had no negative impact on physical and psychosocial functioning [22]. High-performing adolescents and young adults in this specific lifespan might be even more vulnerable for anxiety-related disability based on fear of negative evaluation [23].

The objective of our study is to examine the changes over time of impact of GJH and anxiety on physical and psychosocial functioning with a one-year follow-up within a non-clinical group of high-performing adolescents and young adults.

## 2. Materials and Methods

The HPAA underwent a baseline measurement (T0) at the start of their education at a Dance Academy and a follow-up measurement exactly one year later (T1). This was performed the week before their educational program started. Students were eligible for inclusion when (a) no orthopedic, cardiopulmonary, rheumatological, or neurological problems influencing physical performance were present, and (b) they were able to understand the questionnaires and to adhere to the protocols. The measurements were performed by a team independent of the Dance Academy. Individual feedback on their performance was reported back directly to each student.

Data regarding (loss to) follow-up were collected for HPAA not participating in the follow-up measurement. Possible reasons for loss to follow-up were: (I) unable to participate due to training or logistic problems, (II) unwilling to participate, (III) proceeded their study at a different dance academy or participated in a guest program, and (IV) dropped out based on their performance or due to injury.

The studies were approved by the Medical Ethical Committee (reference numbers W15_093#15.0110 and W16_237#16.277), and written informed consent of all HPAA was obtained.

### 2.1. Procedures

After having received intensive training in the standardized operating procedures during three weeks by expert researchers with broad experience in screening GJH, physiotherapy students performed the functional testing as assessors, and their measurements were analyzed for inter-rater reliability. The assessors performed a specific measurement in both T0 and T1. The expert researchers (JdV, RE) supervised the measurements. The screening performed at T1 was equal to the proceedings and measurements at the start of the study (T0). A detailed description of the measurements is described within the study of de Vries et al. [22].

In brief, measurements started with a visual analog scale combined with a Manikin to evaluate pain and questionnaires assessing injury history, fatigue, and general health status. HPAAs’ joint hypermobility was measured without a warm-up using the Beighton score following completion of questionnaires [24]. Then body composition was measured, followed by muscle strength measurements using a hand-held dynamometer [25] and the Steep Ramp test performed at a standardized protocolled electronically braked cycle ergometer [26]. In between, questionnaires regarding psychosocial parameters of coping (by the Pain coping Scale and the Pain Vigilance and Awareness Questionnaire, PVAQ), anxiety, and depression (by the Hospital Anxiety and Depression Scale, HADS) were completed [27,28,29].

### 2.2. Statistical Analyses

The distribution of the data was checked and combined if normality was confirmed by a Shapiro–Wilk test and visual assessment. Data were presented as means, standard deviations, and ranges, whereas skewed data were presented as median (50th percentile) and range (25th and 75th percentile). Clinical characteristics were stratified for gender. Likewise, to the baseline study, the four subgroups based on GJH and anxiety were used within the analyses. Still, the classification of T0 was repeated at T1, and the results were compared to check for differences in classification with an intraclass correlation coefficient (absolute agreement). Afterward, differences in the baseline for physical and psychosocial parameters were compared between HPAA that participated in the follow-up and HPAA that did not (transferred students, dropped out, or were otherwise unable to participate at the measurements at T1). In order to study the impact of GJH and anxiety over time on those lost to follow-up, a log-linear regression was performed with the dependent variable participation in the second assessment (yes/no). The four subgroups were used as independent variables.

At T1, outcomes were compared between the reference group (HPAA without GJH and without anxiety) and the other three groups (based on the classification of T0) and to cut-offs from normative data (non-dancers). A Bonferroni test was used for multiple testing with a confidence level of 1.67%. Then, the four subgroups were tested on changes within each subgroup between T0 and T1 using a paired T-test or a Wilcoxon signed Rank test and presented as a mean difference (MD) with a standard deviation and a 95% confidence interval (95% CI).

## 3. Results

In total, 101 of the 168 HPAA screened at baseline (T0) participated in the follow-up measurements. Of the 46 HPAA that did not participate, 27 chose to proceed with their study at a different dance academy, 13 dropped out based on their performance, and 6 dropped out due to an injury. Twenty-two were unable to be present at the T1 measurement.

An overview at T1 with changes in physical and psychosocial parameters compared to T0 is presented in Table 1. GJH was present in 28.7% of all HPAA at T1 (33.3% of the females and 18.8% of the males). Anxiety did not change significantly over time and remained present in 52.3% of all HPAA (56.5% females and 43.8% males). Both male and female HPAA had a significant increase in total muscle strength (respectively, males MD (SD) 127 (169), *p* < 0.001, females MD(SD) 55(213), *p* < 0.001) and fatigue (respectively, males MD(SD) 9(19), *p* < 0.05, females MD(SD) 18(19), *p* < 0.001). Pain coping decreased significantly (respectively, males MD(SD) −5(9), *p* < 0.05; females (MD)(SD) −4(8), *p* < 0.001).

When comparing the subgroups at T0 and T1, there was an absolute agreement between the classification of GJH and anxiety at T0 and T1 was 54.7% (*p* < 0.01).

We compared baseline characteristics of the HPAA who missed T1, dropped out, or transferred with the HPAA that were screened at T1. All HPAA that missed T1 had at T0 significantly higher psychosocial scores on anxiety MD(SD) 2(1), *p* < 0.01, and vigilance MD(SD) 8(3), *p* < 0.01 than their peers that were screened at T1. At baseline, the dropouts showed only significantly increased fatigue scores MD(SD) 9 (3), *p* < 0.05 than HPAA that were still enrolled at T1. Transferred students weighted significantly less MD(SD) 4.2(2), *p* < 0.05 and had significantly lower BMI MD(SD) 2.2(0.5), *p* < 0.01 at baseline than HPAA that were still enrolled at T1.

In order to study the impact of GJH and anxiety, the outcomes of physical and psychosocial parameters of the reference group (HPAA without GJH and anxiety) were cross-sectionally compared to the other three groups. The group with GJH and the group with anxiety scored comparable on physical and psychosocial factors to their peers without GJH and anxiety.

HPAA with GJH and anxiety weighted significantly less (respectively, MD(SD) *p*-value 8.6 (2.9), *p* < 0.05) and had less muscle strength (respectively, MD(SD) *p*-value 195 (201), *p* < 0.05) as the reference group but also had almost twice as many females (84.0%).

Within each subgroup, changes in scores (delta) between T0 and T1 were calculated and presented in Table 2.

### 3.1. No GJH/No Anxiety

HPAA without GJH and anxiety showed increased muscle strength and fatigue (respectively, MD(SD) *p*-value 118 (128), *p* < 0.01 and MD(IQR) *p*-value 16 (−28–38), *p* < 0.05) between T0 and T1. All other physical and psychosocial parameters remained stable.

### 3.2. GJH

HPAA with GJH showed significantly decreased Beighton score (respectively, MD(IQR) *p* value-2 (−3–−1), *p* < 0.05) and pain complaints (respectively, MD(SD) *p*-value 41(78), *p* < 0.05), whereas fatigue increased (respectively, MD(IQR) *p*-value 25(16–35), *p* < 0.05) between T0 and T1. All other physical and psychosocial parameters remained stable.

### 3.3. Anxiety

HPAA with anxiety had significantly increased scores of muscle strength and fatigue (respectively, MD(IQR) *p*-value 12 (5–18), *p* < 0.05 and MD(SD) *p*-value 109 (144), *p* <0.01) between T0 and T1. All other physical and psychosocial parameters remained stable.

### 3.4. GJH and Anxiety

HPAA with GJH and anxiety showed increased muscle strength (respectively, MD(SD) *p* value 81 (125), *p* < 0.05), workload (respectively, MD(IQR) *p* value 0.3 (0–0.54), *p* < 0.05) and fatigue (respectively, MD(IQR) *p* value 12 (5–20), *p* < 0.05) between T0 and T1. All other physical and psychosocial parameters remained stable.

### 3.5. Lost to Follow Up

Loglinear regression analysis showed only within HPAA with GJH and anxiety a significantly lower odds ratio, respectively, ß (95% CI) *p*-value 0.4 (0.2–0.9); *p*-value 0.039, on participating in the second assessment. Of all HPAA with GJH and anxiety measured at T0, 55% dropped out before their second year started.

The other groups, HPAA without GJH and anxiety, GJH, and anxiety, showed lower dropout rates of respectively, 34%, 29%, and 33%, with no significant odds on participating in the second assessment.

## 4. Discussion

This follow-up study is the first to show the impact over time of GJH and anxiety in HPAA. Significantly more HPAA with the combination of GJH and anxiety at T0 were not seen at follow-up (T1). Of those HPAA that participated in T1 physical and psychosocial parameters were comparable to all HPAA, independent of the presence of GJH and/or anxiety. All HPAA had a significant increase in their level of fatigue between T0 and T1.

All HPAA that participated in the follow-up assessments showed that the presence of GJH and anxiety separately has no influence on their physical and psychosocial functioning.

However, a remarkable finding was the significantly higher loss to follow-up in the HPAA with both GJH and anxiety [22]. This is supported by the low odds of participation in the second assessment for the group with GJH and anxiety. This finding seems in accordance with earlier findings in the literature stating that HPAA with GJH is struggling to keep up, possibly resulting in more injuries and decreased levels of physical functioning [13,30,31]. However, these studies did not differentiate between individuals with GJH alone and individuals with the combination of GJH and anxiety.

In the present study, those with GJH alone had no increased risk of leaving the dance academy. The prevalence of anxiety in young athletes in the literature was only one-third of the prevalence we found using the same questionnaire. Known barriers to help-seeking for mental health in young athletes are described by Gulliver et al. and consist of not knowing about mental disorders or their symptoms, when to seek help, and being worried about what both the personal and sport environment around the high performer will think [32].

It is important to mention that we conducted this research in a healthy, successful HPAA. All participants were informed about their scores, including the increased anxiety scores after the screening, in a personal report. Still, almost 80% that had anxiety at T0 remained anxious at T1, and HPAA with both GJH and anxiety decreased physical and psychosocial functioning at T0 compared to peers [22]. It is unknown if they reached out for help or consulted a professional for their mental problems, or changed their training routines after T0.

The overall increased fatigue scores were unexpected because the moment of measurement was just before the start of the academic year, after a six-week summer break. Moreover, 40% had a serious risk for sick leave and work disability at T1, again with a higher incidence in HPAA with GJH and anxiety. The demand for HPAA is high with general technical training and time spent to perfection the esthetic part of the performance. Dancers are found to “dance through” their injuries and fatigue and value training and frequent repetition more than recovery time and rest [33]. When there is a negative balance between recovery time and training, HPAA could suffer from non-functional overreaching or overtraining syndrome [34]. Symptoms of overtraining syndrome and non-functional overreaching are characterized by fatigue, performance decline, and mood disturbances which could be a reason for quitting.

The presence of generalized joint hypermobility and psychosocial problems has recently been studied by Meulenbroek et al. [19]. They stated that GJH seems to make individuals more vulnerable to injury and experience musculoskeletal pain more frequently. In addition, a vulnerability for heightened pain-related fear is proposed as an underlying mechanism explaining the relationship between GJH and disability. Thus far, the relation between anxiety and GJH or JHS/EDS has been mostly described within adults [20,35,36,37,38]. This is in accordance with a theoretical model of disease progression by Castori et al. [21,39]. However, our study shows that not only in adults with GJH but already in adolescents and young adults with GJH, clinicians should be aware of the presence of anxiety.

This study has intrinsic limitations that need consideration. First, the T1 assessment took place right before the start of the second year at the dance academy. For HPAA that were not participating at T1, follow-up data were provided by the Dance academy. Students that missed the follow-up assessment but were eligible for starting did not share specific reasons for missing the assessment.

Dancers that chose to continue their dance education at a different dance academy did not share the underlying reasons or information if they continued at the same level or had to repeat the first year, for example. For these dancers that missed the assessment or continued elsewhere, it is not possible to draw any conclusions about their capacity to proceed with dance education. However, comparing the baseline characteristics of all loss to follow-up groups, it is shown that their physical functioning did not differ. The dancers that dropped out of the education might have been already struggling to keep up by having already increased fatigue levels at T0.

Secondly, we questioned the HPAA about their injury history and received treatment. Because this only concerned physical injuries and not their mental health, we do not have any information on whether they consulted a professional or adapted their routine in any way after receiving their personal report reflecting their scores of T0. Despite their awareness of their joint hypermobility, none mentioned a possible mental health issue. Therefore, it is unknown whether the high prevalence of anxiety is persistent due to not receiving help from professionals or not being effectively treated by professionals.

This study provides a clear insight into the physical and psychosocial parameters of HPAA in the starting phase of their dance education. It underlines the importance of a broader baseline screening, including both physical as well as mental aspects of the dancer. Clinicians working with dancers should realize that the combination of GJH with anxiety increases the risk of leaving the dance academy after one year and the presence of only GJH is no liability. Therefore, caregivers and staff should be sensitive to the presence of anxiety in dancers. Additionally, dancers should be informed about the possible consequences of “pushing through” and have access to a safe environment to discuss their anxiety.

## 5. Conclusions

We studied high-performing adolescents and young adults (HPAA) and the changes over time of impact of Generalized Joint Hypermobility and anxiety on physical and psychosocial functioning with one-year follow-up and found in 52.3% that anxiety did not change significantly over time, whereas GJH was present in 28.7%. Fatigue increased significantly in all HPAA at one-year follow-up. A significantly lower odds ratio for participating in the second assessment was present in HPAA with GJH and anxiety with a 55% dropout rate after one year. This confirms the segregation between GJH combined with anxiety or GJH alone. The fatigue levels of all HPAA increased significantly over time to a serious risk for sick leave and work disability. This study confirms the association between GJH and anxiety but especially emphasizes the disabling role of anxiety. Screening for anxiety is relevant in HPAA with GJH and might influence tailored interventions.

## Figures and Tables

**Table 1:** Clinical characteristics at T0, T1, and difference scores for all dancers that were assessed at both measurements.

	Males (*N* ^a^ = 32)						Females (*N* = 69)					
	T0		T1		Difference MD (SD) 95% CI ^b^		T0		T1		Difference MD (SD) 95% CI	
Clinical Characteristics												
**Age**	20 (3)	16–28	21 (3)	17–28	0.5 (0.4)	0.3–0.6	19 (3)	16 -30	20 (3)	16–30	0.4 (0.9)	0.2–0.6
**Weight, kg** **^c^ (mean, SD, range)**	67.8 (7.9)	54–91	71.4 (8.4)	58–95	2.4 ** (3.5)	1.1–3.7	57.3 (8.4)	39–100	59.7 (8.3)	46–100	1.1 * (2.7)	0.5–1.8
**BMI, kg/m^2^** **^d^ (mean, SD, range)**	21.3 (2.0)	18–29	22.4 (2.2)	15–19	0.7 * (1.7)	0.1–1.3	20.9 (2.7)	16–30	21.9 (3.0)	17–34	0.5 * (1.6)	0.1–0.9
**Joint hypermobility: Beighton (median, 25th and 75th percentile)**	3	0–7	2	0–7			5	0–9	3	0–9		
**Beighton** **(*N, %*** **^e^** **)**	7	21.9%	6	18.8%			37	53.6%	23	33.3%		
**Pain; VAS, mm (median, 25th and 75th percentile)**	17	0–303	28	0–241			11	0–347	1	0–277		
**Presence of an injury last year** **(*N*, %)**	10	31.2%	13	40.6%			23	33.3%	15	21.7%		
**Psychosocial Characteristics**												
**Fatigue total score (median, 25th and 75th percentile)**	51	23–89	58	27–86			50	21–89	75	26–95		
**Anxiety (median, 25th and 75th percentile)**	7	1–14	7	2–13			9	0–14	8	2–16		
**Anxiety disorders (*N*, %)**	15	46.9%	14	43.8%			42	60.9%	39	56.5%		
**Depression (median, 25th and 75th percentile)**	4	0–14	5	0–14			4	0–10	3	0–13		
**Catastrophizing, PCS ^f^ total (median, 25th and 75th percentile)**	11	0–40	11	0–26			11	0–38	7	0–35		
**Vigilance, PVAQ ^g^ total (median, 25th and 75th percentile)**	39	7–60	35	20–65			34	12–58	36	7–64		
**Physical Characteristics**												
**Workload, Wrpeak (W/kg ^h^), (mean, SD, range)**	6.0 (0.7)	4.4–7.5	6.2 (0.8)	5.1–7.9	0.2 (0.8)	−0.1–0.5	5.2 (0.8)	2.5–7.2	5.3 (1.1)	0–7	0.0 (1.1)	−0.3–0.3
**Total muscle strength (Newton) (mean, SD, range)**	1844 (174)	1525–2263	1977 (190)	1423–2426	130 ** (165)	71–190	1384 (170)	889–1699	1478 (247)	0–1932	59 * (213)	8–110

* *p* < 0.05; ** *p* < 0.001 *p* values test differences within individuals in groups between T0 and T1. ^a^ *N* Number of participants, ^b^ MD (SD) 95% CI Mean Difference (Standard Deviation) 95% Confidence Interval, ^c^ kg kilogram, ^d^ m meter, ^e^ % percentage, ^f^ PCS “Pain Catastrophizing Scale” total score 0–52, ^g^ PVAQ “Pain Vigilance and Awareness Questionnaire” total score 0–80, ^h^ Wrpeak (W/kg) peak workload in wattage per kilogram.

**Table 2:** Changes (delta) in psychosocial and physical outcomes between T0 and T1 scores within dancers with Generalized Joint Hypermobility and/or anxiety, or no GJH and anxiety presented as mean difference, standard deviation and 95% confidence interval.

	All (*N* ^a^ = 101)											
	None (*N* = 25)			GJH (*N* = 19)			Anxiety (*N* = 32)			GJH and Anxiety (*N* = 25)		
**Weight, kg ^b^**	2.2 *	(3.6)	1–4	1.1	(2.7)	0–2	1.4 *	(2.8)	0.4–2.4	1.4 *	(3.0)	0.2–2.7
**BMI, kg/m^2^**	0.8 *	(1.4)	0–1	0.3	(1.9)	−1–1	0.9 *	(1.7)	0–1	0.2	(1.4)	−0.4–0.8
**Joint hypermobility, Beighton**	0	(2)	−1–1	−2 *	(2)	−3–−1	0	(2)	−1–1	−1	(3)	−2–0
**Pain, VAS, mm**	5	(81)	−28–38	41 *	(78)	3 - 79	−14	(78)	−42–15	15	(69)	−13–44
**Psychosocial Characteristics**												
**Fatigue, CIS20 ^c^ total score**	16 *	(21)	−28–38	25 *	(20)	16–35	12 *	(18)	5–18	12 *	(19)	5–20
**Anxiety**	0	(3)	−1–2	2 *	(3)	0–4	−1	(3)	−2–0	0	(2)	−1–0
**Depression**	0	(2)	−1–1	0	(2)	−1–1	1	(3)	−1–2	−1	(4)	−2–1
**Catastrophizing, PCS ^d^ total**	−4 *	(8)	−7–0	−2	(8)	−6–2	−3	(9)	−6–1	−3	(10)	−7–1
**Vigilance PCS ^d^ total?**	−1	(12)	−6–4	1	(10)	−4–6	−1	(9)	−4–3	2	(11)	−3–6
**Physical Characteristics**												
**Workload, Wrpeak ^e^, W/kg**	−0.1	(0.9)	−0.5–0.2	0.1	(1.9)	−5.1–4.7	0	(0.7)	−0.3–0.2	0.3 *	(0.6)	0.0–0.54
**Total muscle strength, Newton**	118 **	(126)	66–170	−12	(368)	−1385–364	109 **	(144)	57–160	81 *	(125)	29–133

* *p* < 0.05; ** *p* < 0.001 *p* values test differences within individuals in groups between T0 and T1. ^a^ *N* Number of participants, ^b^ kg kilogram, ^c^ CIS 20 “Checklist Individual Strength” total score 20–140, ^d^ PCS “Pain Catastrophizing Scale” total score 0–52, ^e^ Wrpeak (W/kg) peak workload in wattage per kilogram.

## Data Availability

The data presented in this study are available on request from the corresponding author. The data are not publicly available due to privacy restrictions.
